# Proteomics profiles of blood glucose-related proteins involved in a Chinese longevity cohort

**DOI:** 10.1186/s12014-022-09382-w

**Published:** 2022-12-03

**Authors:** Rong Zhang, Fengjuan Liu, Shengliang Ye, Xi Du, Li Ma, Haijun Cao, Zongkui Wang, Changqing Li

**Affiliations:** Institute of Blood Transfusion, Chinese Academy of Medical Sciences & Peking Union Medical College, Chengdu, 610052 China

**Keywords:** TMT, Proteomics, Fructosamine, Longevity, Blood glucose

## Abstract

**Background:**

High blood glucose level is one of the main characteristics of diabetes mellitus. Based on previous studies, it is speculated longevity families may have certain advantages in blood glucose regulation. However, limited information on these items has been reported. The purpose of this study was to profile differences of plasma proteomics between longevity subjects (with normal fructosamine (FUN) level) and non-longevity area participants (with exceeding standard FUN level).

**Methods:**

In this study, a TMT-based proteomics analysis was used to profile differences of plasma proteomics between longevity subjects (with normal FUN level) and non-longevity area participants (with exceeding standard FUN level). Results were validated by Luminex detection.

**Results:**

A total of 155 differentially expressed proteins (DEPs) were identified between these two groups. The DEPs related to blood glucose regulation were mainly involved in glycolysis/gluconeogenesis, pyruvate metabolism and propanoate metabolism, and most of the DEPs were contained in carbohydrate metabolism, PI3K-Akt pathway, glucagon signaling pathway and inflammatory response. Validation by Luminex detection confirmed that CD163 was down-regulated, and SPARC, PARK 7 and IGFBP-1 were up-regulated in longevity participants.

**Conclusions:**

This study not only highlighted carbohydrate metabolism, PI3K-Akt pathway, glucagon signaling pathway and inflammatory response may play important roles in blood glucose regulation, but also indicated that YWHAZ, YWHAB, YWHAG, YWHAE, CALM3, CRP, SAA2, PARK 7, IGFBP1 and VNN1 may serve as potential biomarkers for predicting abnormal blood glucose levels.

**Supplementary Information:**

The online version contains supplementary material available at 10.1186/s12014-022-09382-w.

## Background

Diabetes mellitus (DM), a serious, chronic disease, is one of the top 10 causes of death globally. The estimate of global diabetes prevalence in the 20–79 year age group was tripled from 151 million in 2000 to 537 million in 2021. And about 6.7 million people aged 20–79 years are estimated to die from diabetes-related causes in 2021 [1]. People living with diabetes have high risk of serious complications, which leading to undue stress on medical systems, society and families [1, 2].

DM usually occurs when blood levels of glucose raise. Blood glucose can be monitored by fasting plasma glucose (FPG) value, the 2-h plasma glucose (2-hPG) value during a 75-g oral glucose tolerance test (OGTT), or glycated haemoglobin (HbA1c) criteriaion. Other biochemical markers, such as 1, 5-anhydroglucitol (1, 5-AG), glycatedalbumin, FUN, adiponectin, C-peptide are also used in diagnosing and monitoring DM [2–4]. Among them, FUN can provide information about glycemic levels over a period of two to 3 weeks. It can be used to monitor DM, particularly in cases with restrictions on the use of glycated hemoglobin [5].

Bama Yao Autonomous County, which located in Guangxi Zhuang Autonomous Region of China, is one of “The World’s Longevity Town ship” [6]. The report of International Diabetes Federation (IDF) showed that the prevalence of diabetes increases with age [1]. Similar results have been confirmed in populations of Bama. But it is interesting to note that the average blood glucose level in Bama area is lower than that in other regions, and the blood glucose level of longevity family group of Bama is significantly lower than that of non-longevity family group [7–9]. Therefore, it is speculated that longevity families may have certain advantages in blood glucose regulation. But till now, limited information on these items was reported.

In our previous studies [6, 10], we found some differentially expressed proteins (DEPs) between Bama longevous family group and normal controls from non-longevous region of China. Some of these DEPs such as PGK1, ENO1, LDHA, LDHB, PKM, and GAPDH, were glycolysis/gluconeogenesis-related proteins. Otherwise, GCK and ARL2BP, which were the specifically and differentially expressed autoantibodies in the offspring from longevous and non-longevous families from Bama, were related to the regulation of blood glucose. To further investigate the characteristics of blood glucose regulation in Bama-induced longevity, Tandem mass tag (TMT)-based approach was used to systematically explore the expression of plasma proteins in the plasma donors from non-longevous region which have an exceeding standard FUN level and offspring of longevous families in Bama which have normal FUN level in this study. The DEPs were revealed by bioinformatics analysis and human magnetic Luminex screening assay was used to verify the result of TMT-based analyses.

## Methods

The workflow of the present study was shown in Additional file 1: Figure S1.

### Sample collection

Two groups were enrolled in this study. A total of 30 participants of group A were enrolled from a non-longevous region of China (Shimen, Hunan province), with exceeding standard FUN level (> 286 μmol/L) and were offsprings of non-longevous families (a family with none ≥ 90 years old immediate family members). The volunteers of group B permanently reside in Bama longevity hotspot (Bama, Guangxi province) and were offsprings of longevous families (a family with at least two ≥ 90 years old immediate family members) and with normal FUN level (0.0–286 μmol/L). The ABO, age and gender were matched in group A and B.

According to the standard of the “whole blood and component donor selection requirements” previously described [11, 12], the inclusion criteria were that all participators were over 18 years of age, healthy, and unrelated; the exclusion criteria were people had history of thrombus or hemorrhage, usage of oral anticoagulation therapy, pregnancy, HBV/HCV/HIV infection, hepatic disease, et al.

### Sample preparation

Each participant’s blood sample was collected by plasma apheresis and then immediately stored at −70 °C in aliquots until being transported to institute of blood transfusion (IBT) at Chengdu on dry ice. Once arrived the laboratory, one aliquot of each sample was immediately used for FUN detection, and other aliquots were stored at −70 °C until analysis. The information of participants was shown in Table 1.

**Table 1:** Sample grouping information of proteome analysis of this study^※^

Cohort	Group A(n = 30)			Group B (n = 30)		
	A1 (n = 10)	A2 (n = 10)	A3 (n = 10)	B1 (n = 10)	B2 (n = 10)	B3 (n = 10)
Age (y)	49.50 (41–54)	49.60 (42–55)	49.60 (43–56)	49.30 (36–56)	49.10 (32–57)	49.00 (39–57)
Gender						
Female	6	6	6	5	5	5
Male	4	4	4	5	5	5
Blood type						
A	3	3	3	3	3	3
B	2	2	2	2	2	2
AB	1	1	1	1	1	1
O	4	4	4	4	4	4
FUN (μmol/L)^**^	356.55 ± 46.35	387.66 ± 64.04	351.56 ± 63.72	186.67 ± 23.10	190.28 ± 26.53	179.01 ± 13.20
TG(mmol/L)^**^	3.34 ± 1.45	3.67 ± 1.64	2.93 ± 0.96	1.61 ± 1.43	1.20 ± 0.40	1.34 ± 0.59
TC(mmol/L)	3.99 ± 0.57	4.61 ± 1.02	3.87 ± 0.71	4.17 ± 0.89	4.17 ± 0.48	4.54 ± 0.72
HDLC(mmol/L) ^**^	0.74 ± 0.15	0.84 ± 0.31	0.80 ± 0.15	1.21 ± 0.37	1.13 ± 0.26	1.12 ± 0.22
LDLC(mmol/L)	2.29 ± 0.47	2.69 ± 0.77	2.06 ± 0.42	2.32 ± 0.60	2.49 ± 0.33	2.83 ± 0.76
APOA1(g/L)^**^	1.09 ± 0.17	1.20 ± 0.20	1.13 ± 0.14	1.46 ± 0.22	1.48 ± 0.24	1.48 ± 0.23
APOB(g/L)	0.70 ± 0.17	0.84 ± 0.20	0.67 ± 0.17	0.65 ± 0.24	0.83 ± 0.22	0.84 ± 0.23

Normal value range: FUN (0–286 μmol/L), TG (0.0–2.3 mmol/L), TC (0.0–5.6 mmol/L), HDLC (≥ 0.9 mmol/L), LDLC (0.0–4.11 mmol/L), APOA1(1.1–1.7 g/L), APOB(0.66–1.33 g/L)

^**※**^Group A, participants from a non-longevity area, with an exceeding standard FUN level; Group B, offsprings of longevous families, with normal FUN level

Group A and Group B were conducted by two-tailed unpaired Student’s *t* test. ^*^p < 0.05, ^**^p < 0.01

### Sample preparation, TMT-labelling, HPLC fractionation and LC–MS/MS

For TMT-based proteomic analysis, 30 plasma samples of each cohort were marked as Group A and Group B, respectively. Pooled plasma samples were obtained by mixing equal volumes of each 10 individual plasma samples from each group. Then a ProteoMiner™ Protein Enrichment Introductory Large-Capacity Kit (Bio-Rad, Richmond, USA) was used to remove the high abundance proteins of the pooled plasma. Total protein concentrations of the low abundance protein enrichment plasma samples were determined using a bicinchoninic acid (BCA) protein assay kit (Pierce, Rockford, IL, USA), and SDS-PAGE was used to verify the consistency of each group.

Subsequently, protein digestion and TMT-labeling were done with the detailed procedures in our previous studies [6, 10]. In short, approximately 100 μg proteins per sample were digested by a procedure of two-step tryptic digestion after samples were reduced and alkylated according to the protocol. Then each group of digested peptides was labeled using a 6-plex TMT labeling kit (Thermo Fisher Scientific, Torrance, CA, USA). Sample labeling was as follows: A1:126, A2:127, A3:128, B1:129, B2:130, and B3:131.

Thereafter, high pH reverse-phase high-performance liquid chromatography (HPLC) was used to fractionate the labeled samples into 60 fractions. Then, the peptides were combined into 18 fractions and vacuum-dried. The dried samples were subsequently reconstituted and tested by LC-MS/MS.

Afterwards, using an EASY-nLC 1000 UPLC system (Thermo Fisher Scientific, San Jose, USA), the peptides from each fraction were dissolved in 0.1% formic acid, directly loaded onto a reversed-phase analytical column (150 mm length, 75 μm ID). The gradient was comprised of an increase from 7 to 25% solvent B (0.1% formic acid in 90% acetonitrile) over 26 min, 25–40% in 8 min, climbing to 80% in 3 min, and then holding at 80% for the last 3 min, at a constant flow rate of 400 nL/min. Then the peptides were analyzed by tandem mass spectrometry (MS/MS) using Q Exactive Plus (Thermo Fisher Scientific). The electrospray voltage applied was 2.0 kV. Intact peptides and ion fragments were detected in the orbitrap at a resolution of 70,000 and 17,500, respectively. In the MS survey scan, a data-dependent mode with an automatic alteration (1 MS scan followed by 20 MS/MS scans) was used for the top 20 precursor ions above a threshold ion count of 5 × 104 with 30 s dynamic exclusion. The m/z scan range was 350–1800, and the fixed first mass was set as 100 m/z. Automatic gain control (AGC) was used to prevent overfilling of the Orbitrap.

### LC–MS/MS data and bioinformatics analysis

The MS/MS data were processed by Max Quant search engine (v.1.5.2.8) against human UniProt database (http://www.uniprot.org; Taxon ID 9606, 20,380 entries). Trypsin/P was specified as cleavage enzyme allowing up to 2 missing cleavages. The precursor mass tolerance was set as 20 ppm in First search, 5 ppm in Main search, and the mass tolerance for fragment ions was set as 0.02 Da. Acetylation (Protein N-term) modification and oxidation on Met were specified as variable modifications, and carbamidomethyl on Cys was specified as fixed modification. Quantitation method set to TMT 6-plex, and False discovery rate (FDR) was adjusted to < 1% at protein, peptide and PSM levels, and minimum score for peptides was set > 40.

The DEPs were annotated through Gene Ontology (GO) from the UniProt-GOA database (http://www.ebi.ac.uk/GOA/). WoLF PSORT (version of PSORT/PSORT II) and SubLoc (http://www.bioinfo.tsinghua.edu.cn/SubLoc/) were used to predict subcellular localization of DEPs, InterProScan based on protein sequence alignment method (http://www.ebi.ac.uk/interpro/) was used to describe the domain functional of the DEPs, and the Clusters of Orthologous Groups (COG) of protein database was carried out for functional classification of DEPs. The pathway enrichment analysis of the DEPs was assessed by Kyoto Encyclopedia of Genes and Genomes (KEGG) pathway database (http://www.genome.jp/kegg/). And the protein-protein interaction (PPI) net-works were identified and visualized by STRING database (http://string-db.org). A *p*-value < 0.05 was used as the threshold to determine the significant enrichments of GO annotation and KEGG pathways.

### Validation of proteomics results (Luminex liquid suspension chip detection)

Group A (37 individuals from a non-longevous region, with exceeding FUN level) and group B (37 Bama volunteers of longevous families, with normal FUN level) provided blood samples to verify the quantitative data obtained by TMT proteomics. The subjects of validation were all different from the groups of 30 subjects studied in the discovery phase. The information of participants of validation set was shown in Table 2.

**Table 2:** Sample grouping information of Luminex assay of this study*

Cohort	Group A(n = 37)	Group B (n = 37)
Age (y)	49.49 (18–58)	45.77 (25–56)
Gender		
Female	19	21
Male	18	16
Blood type		
A	15	6
B	8	7
AB	0	3
O	14	21
FUN (μmol/L)	366.11 ± 72.49	186.88 ± 43.68

^*^ Group A, participants from a non-longevity area, with an exceeding standard FUN level; Group B, offsprings of longevous families, with normal FUN level

The plasma levels of SPARC (secreted protein acidic and rich in cysteine), CD163 (Scavenger receptor cysteine-rich type 1 protein M130), PARK 7 (Protein/nucleic acid deglycase DJ-1) and IGFBP-1(insulin-like growth factor-binding protein 1) were measured by Human Premixed Multi-Analyte Kit (4-plex; LXSAHM-04, R&D Systems, Inc., Minneapolis, USA). And the assay was performed by Wayen Biotechnologies (Shanghai, China). Briefly, fifty microliters of standards or samples were added into the 96-well polystyrene microplate. Subsequently, fifty microliters of magnetic beads were added and the plate was incubated in dark, with gently shaking at room temperature for two hours. The plate was carefully washed 3 times, and fifty microliters of biotin-antibody were added in each well. The plate was incubated for 1 h and then was washed 3 times. After that, fifty microliters of Streptavidin-PE were added into each well and then the plate was incubated for 30 min. After the final washing step, the values were read by using a Luminex 200 system (Luminex Corporation, Austin, TX, USA), and data were processed by the software (Luminex xPONENT) of the instrument.

## Results

### Overview of protein identification and quantification

**Fig.1:**
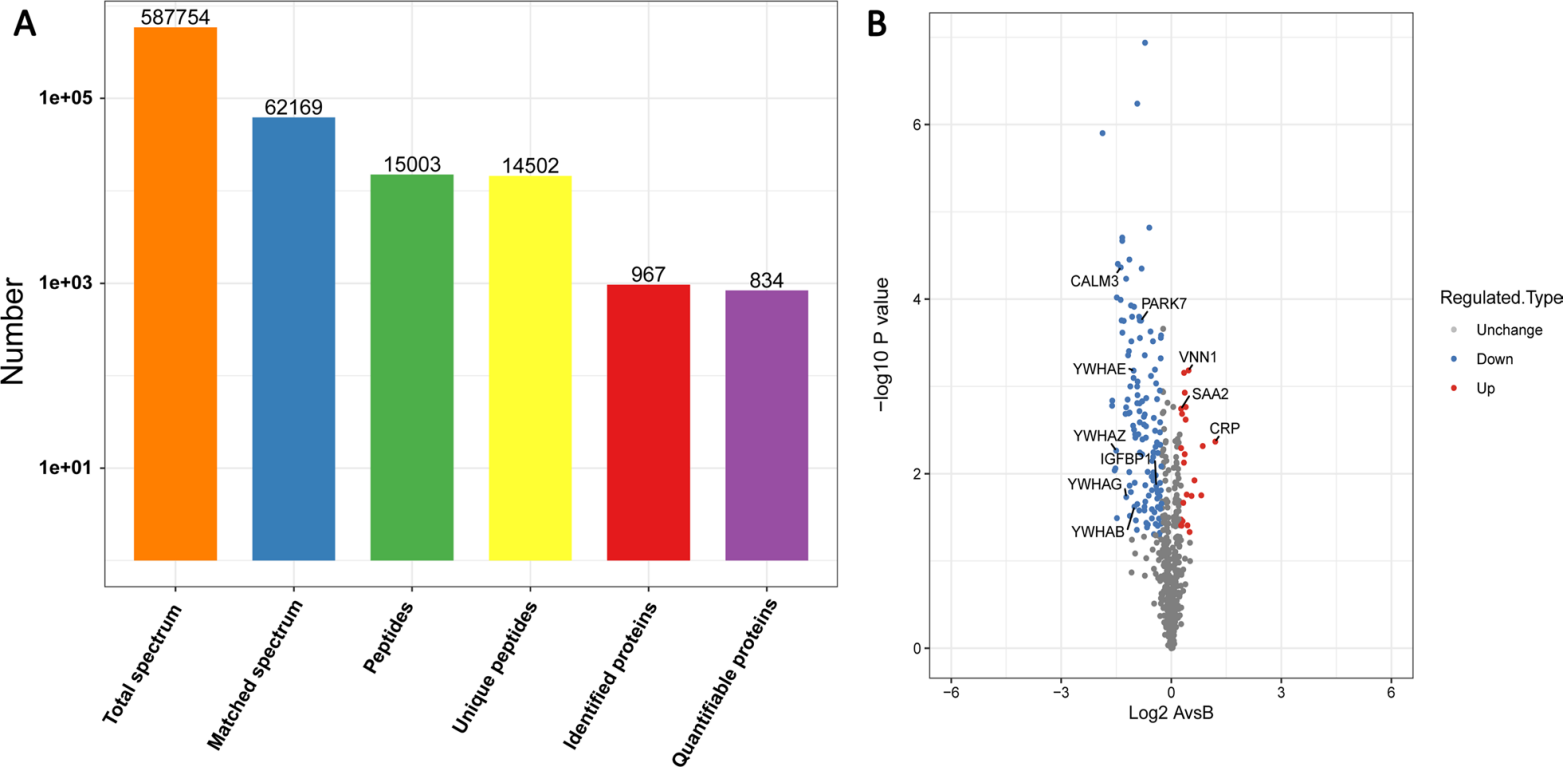
Basic statistics of mass spectral data results. **A** Results of the LC–MS/MS for the proteins. **B** The volcano plot shows the up- (red) or down regulated (blue) proteins between group A and group B

A total of 967 proteins were identified, of which 834 proteins contained quantifiable information (quantified at more than a 95% CI and with no less than two unique peptides) (Fig. 1A). The detailed data of subcellular localization, GO-based annotation, KEGG pathways and domain prediction of the 967 identified proteins are shown in Additional file 2: Table S1. For comparison between group A (non-longevous region participants) and group B (Bama longevous region participants), a protein exhibiting a fold change of > 1.2 or < 0.83 and a *p* value of < 0.05 was regarded as a differentially expressed protein (DEP). Based on these two criteria, 155 DEPs were identified, of which 23 were significantly up-regulated and 132 were down-regulated in group A compared with group B (Fig. 1B and Additional file 3: Table S2).

### Annotation analysis of the differentially expressed proteins

**Fig. 2:**
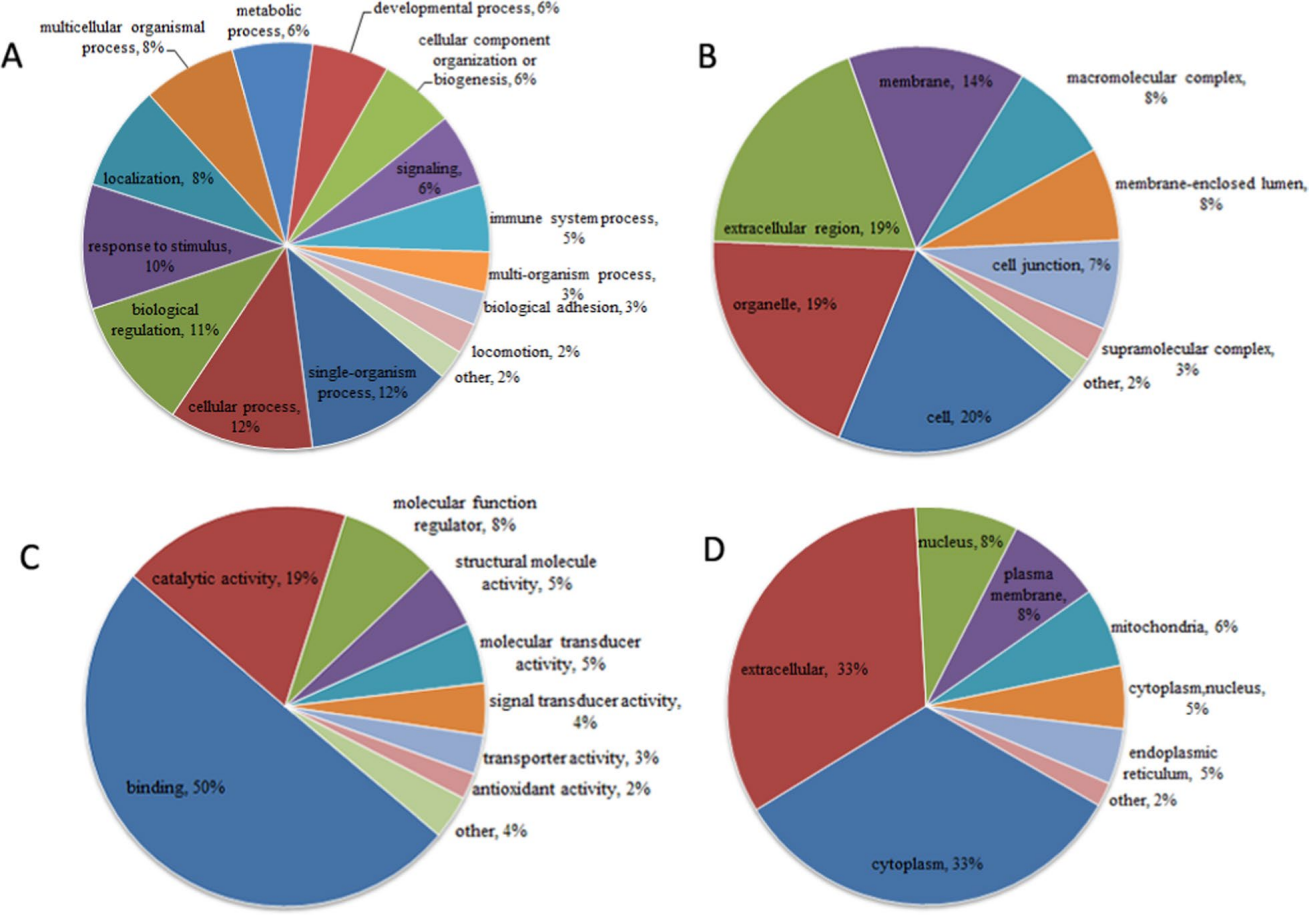
Annotations of the differentially expressed proteins. **A** biological processes, **B** cellular components, **C** molecular functions, and **D** Wolfpsort-based subcellular localization prediction

The 155 DEPs were mainly clustered into 33 GO functional categories, of which accounted to 15 biological processes, 9 cellular components, and 9 molecular functions (Fig. 2 and Additional file 4: Table S3). Biological process analysis showed most of the DEPs were involved in single-organism process (12%, n = 137), cellular process (12%, n = 133), biological regulation (11%, n = 122), and response to stimulus (10%, n = 115) (Fig. 2A and Additional file 4: Table S3). For cellular components, the DEPs were mainly originated from cell (20%, n = 141), organelle (19%, n = 136), extracellular region (19%, n = 133), and membrane (14%, n = 99) (Fig. 2B and Additional file 4: Table S3). The prevalent molecular functions were binding (50%, n = 143) and catalytic activity (19%, n = 53) (Fig. 2C and Additional file 4: Table S3).

In addition, the subcellular localization of DEPs analysis results indicated that cytoplasm (33%), extracellular (33%) and nucleus (8%) were the top three significant subcellular location sites (Fig. 2d, and Additional file 5: Table S4).

**Fig. 3:**
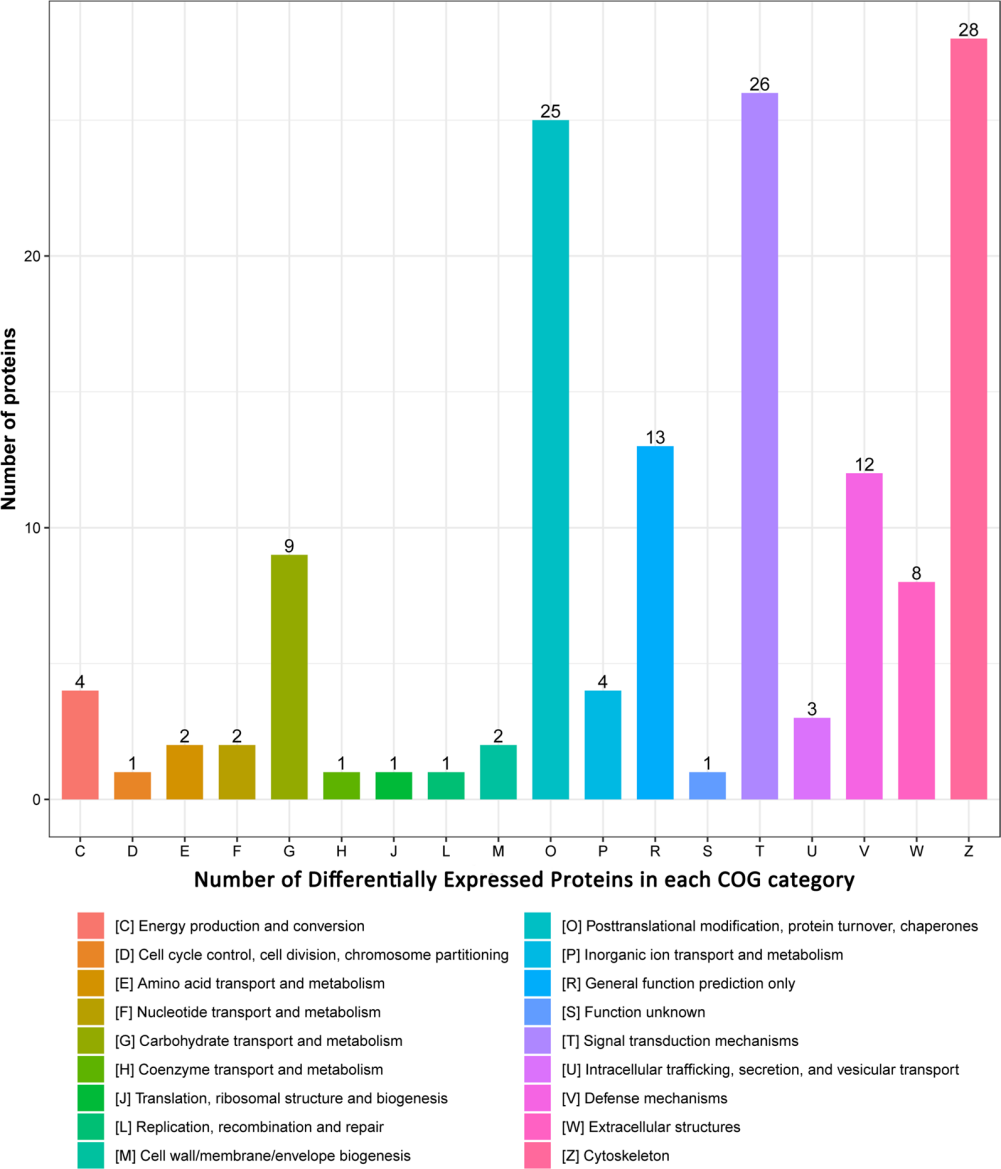
COG analysis of the differentially expressed proteins. The different function DEPs (participants with exceeding standard FUN level from non-longevity area/Bama participants with normal FUN level fold-change > 1.2 or < 0.83) were classified according to GOC database

To further understand the function of the proteins, COG of protein database was carried out for functional classification of DEPs. The results revealed that the DEPs were classified into 18 COG categories, among which, cytoskeleton, signal transduction mechanisms and posttranslational modification, protein turnover, chaperones were the three largest groups. Furthermore, general function prediction only and defense mechanisms also contained many DEPs (Fig. 3, and Additional file 6: Table S5).

**Fig. 4:**
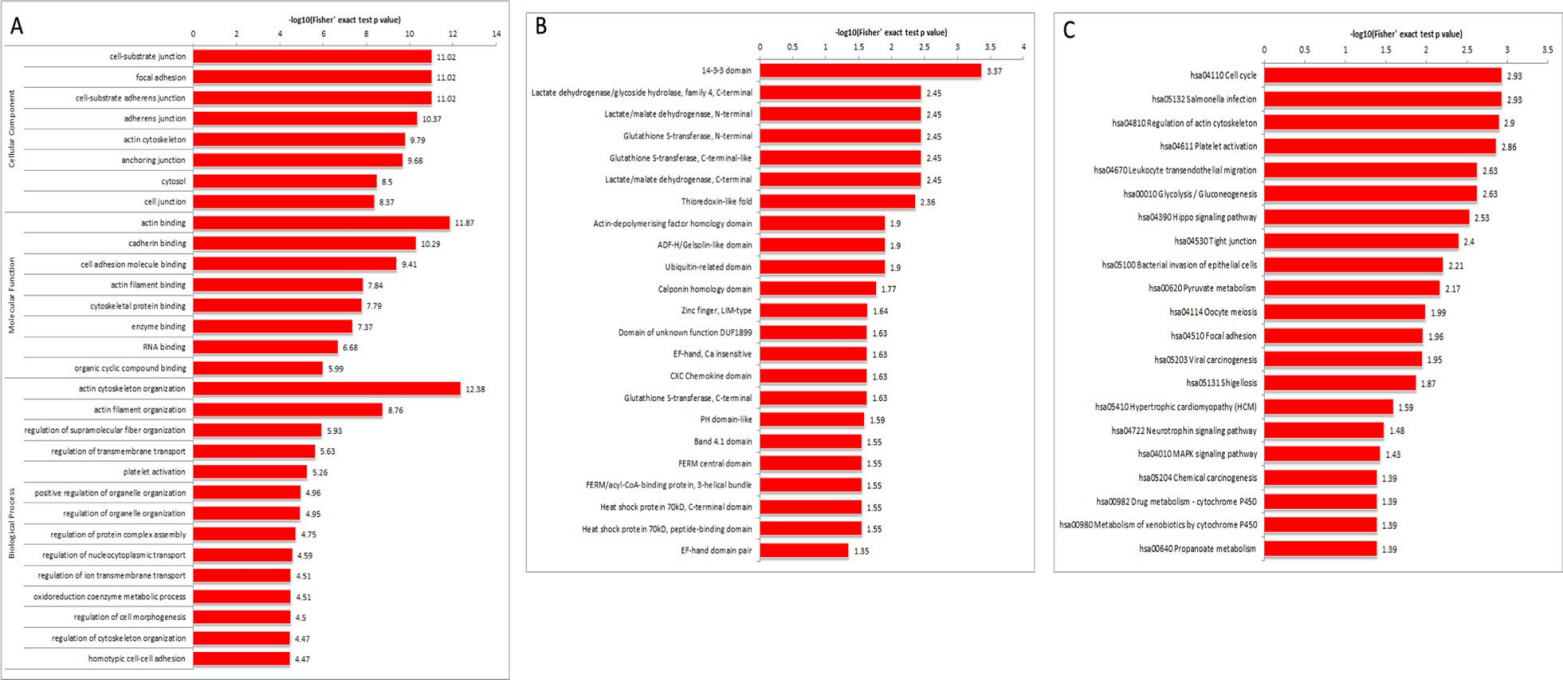
Enrichment analysis of the differentially expressed proteins. **A** GO term enrichment analysis of the DEPs. **B** Protein domain prediction enrichment analysis of the DEPs. **C** KEGG pathway enrichment analysis of the DEPs

GO enrichment analysis is shown that the most significantly enriched cellular components were cell-substrate junction and focal adhesion. The main molecular functions were actin binding, cadherin binding and cell adhesion molecule binding. And the biological processes were mainly enriched in actin cytoskeleton organization and actin filament organization. (Fig. 4A and Additional file 7: Table S6). GO enrichment analysis exhibited that the DEPs were mainly involved in binding, organization, regulation, etc.

The analysis of protein domain enrichment (Fig. 4B and Additional file 8: Table S7) revealed that the most conspicuously enriched term was 14–3-3 domain. It should be noted that most of the significantly enriched domains are directly related to the enzymes that play a role in carbohydrate metabolism, such as “14-3-3 domain”, “Lactate dehydrogenase/glycoside hydrolase family 4, C-terminal”, “Lactate/malate, N-terminal”, and “Lactate/malate dehydrogenase, C-terminal”. This implied that compared with individuals from non-longevous region with an exceeding FUN level, the proteins/enzymes containing the above domains in the plasma of offsprings of Bama longevous families with normal FUN level were significantly changed.

According to KEGG pathway analysis, 21 KEGG pathways were clustered, among them, the top four enriched KEGG pathways were cell cycle, salmonella infection, regulation of actin cytoskeleton and platelet activation (Fig. 4C and Additional file 9: Table S8). Additionally, KEGG pathways which related to carbohydrate metabolism include glycolysis/gluconeogenesis, pyruvate metabolism and propanoate metabolism.

**Fig. 5:**
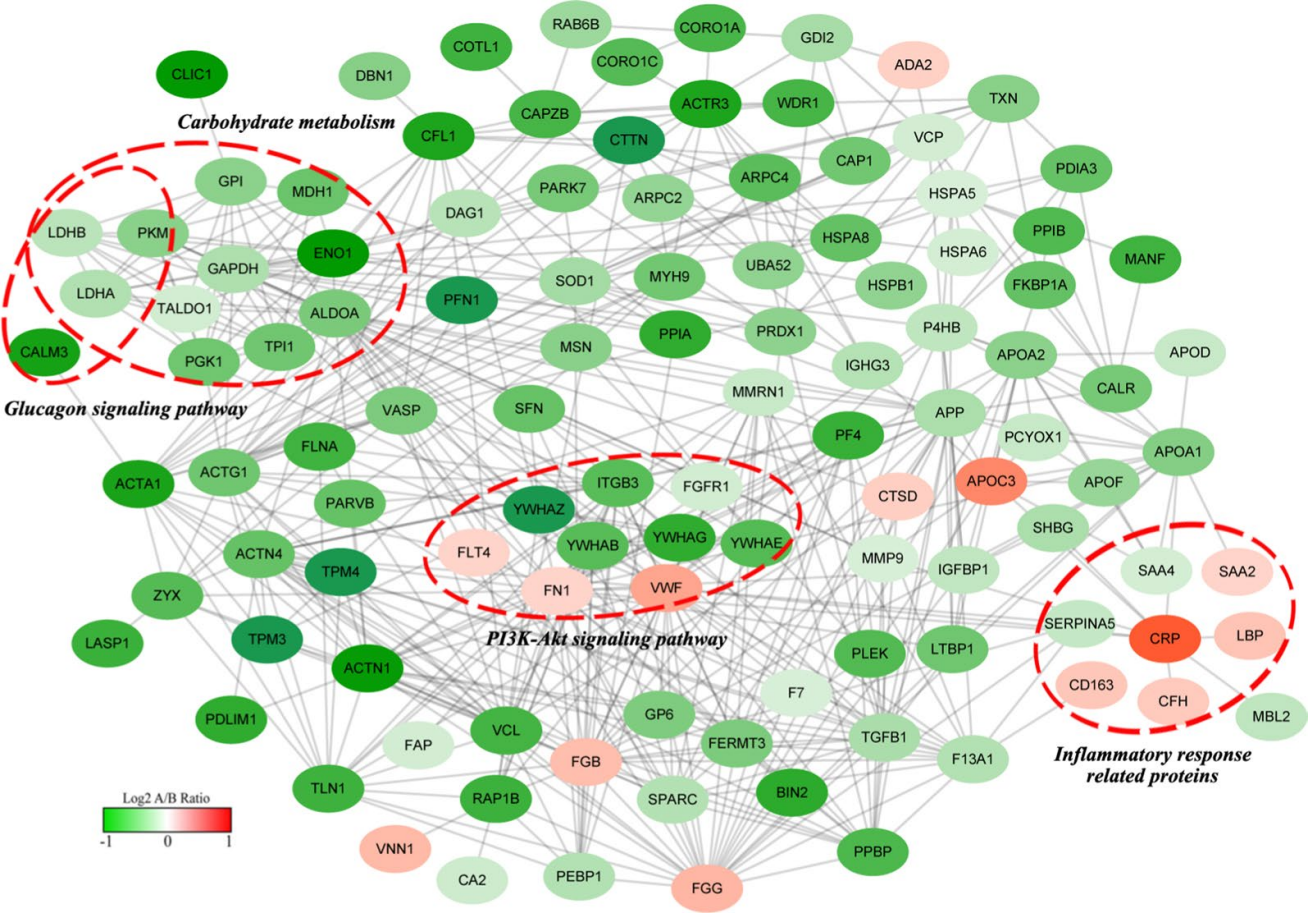
The protein–protein interaction network analysis of the DEPs. The functional interactions of all the 155 DEPs (participants with exceeding standard FUN level from non-longevity area/Bama participants with normal FUN level fold-change > 1.2 or < 0.83) were analyzed by using STRING database Cytoscape software

In order to better understand the interaction among the DEPs, protein-protein interaction (PPI) network analysis by using STRING database and Cytoscape software was subsequently constructed. It obviously revealed that at least three crosstalk signaling clusters which extremely related to regulation of blood glucose were displayed in the complex PPI network, including carbohydrate metabolism, phosphatidylinositol 3-kinase (PI3K) -Akt signaling pathway and glucagon signaling pathway related proteins (Fig. 5).

### Validation of proteomics results with luminex liquid suspension chip detection

**Fig. 6:**
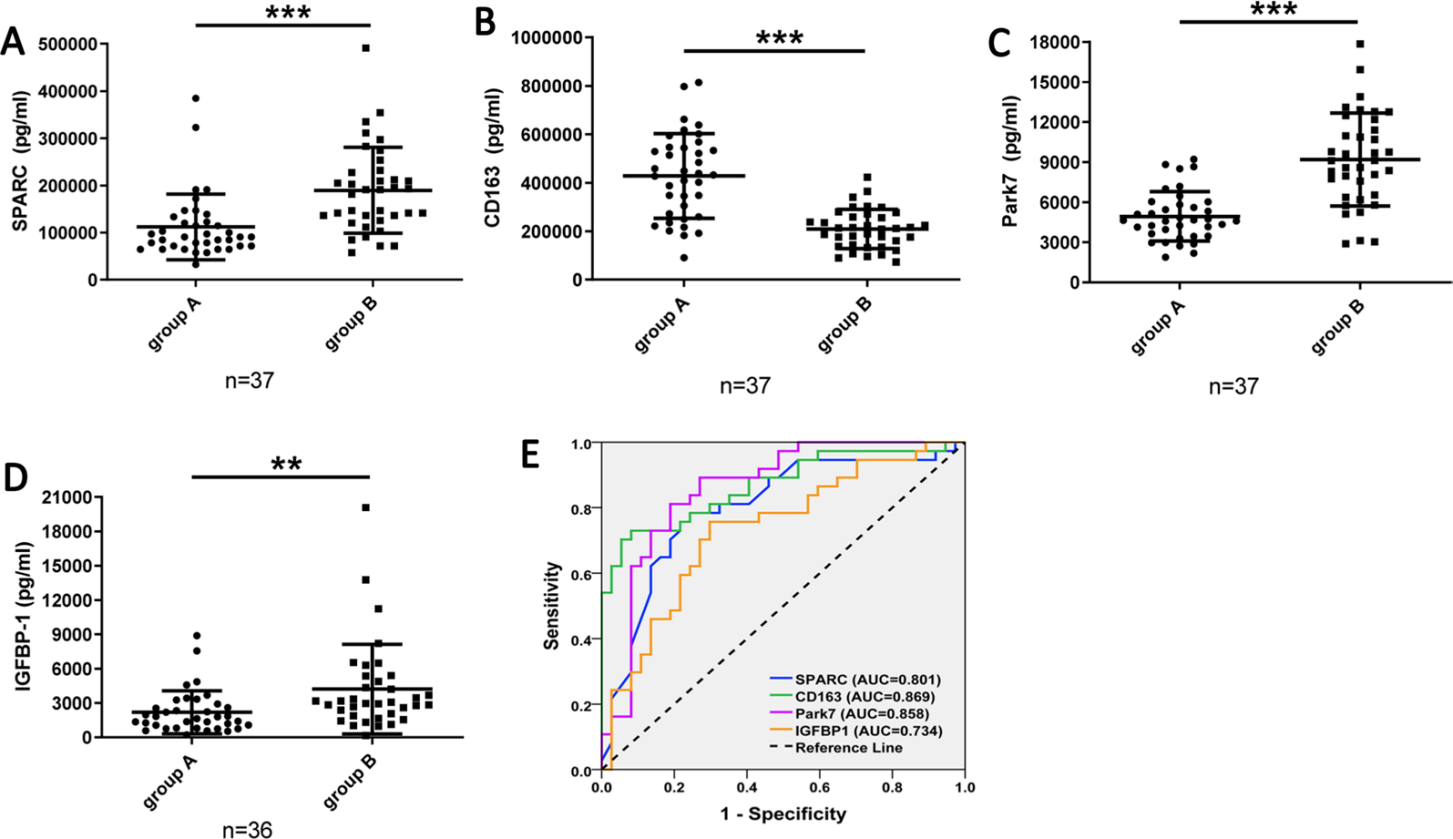
Verification of differentially expressed proteins by human magnetic Luminex screening assay. **A** SPARC, **B** CD163, **C** Park 7, and **D** IGFBP1. Data represent the mean ± SEM for group A (participants with exceeding FUN level from non-longevity area) and group B (Bama participants with normal FUN level). **E** ROC curve analysis of SPARC, CD163, Park7, and IGFBP1 to discriminate participants with an exceeding standard FUN level from participants with normal FUN level

To validate proteomics results of TMT, we selected four proteins involved in different pathways or biological processes, including SPARC, CD163, PARK7 and IGFBP-1 to be validated by human magnetic Luminex screening assay. All of these proteins were significantly up or down regulated (fold change > 1.2 or < 0.83, and *p* < 0.05). As shown in Fig. 6, consistent with the TMT-based proteomics results, plasma levels of CD163 were significantly lower in Bama participants (with normal FUN level, group B) as compared to that in participants from non-longevity area (with exceeding standard FUN level, group A). Whereas plasma levels of SPARC, PARK 7, and IGFBP-1 increased notably in Bama individuals compared with controls. ROC curves were constructed using the validation data, area under the ROC curve (AUC) values of SPARC, CD163, Park7, and IGFBP1 were 0.801 (95% CI 0.696–0.905, *p* < 0.0001), 0.869 (95% CI 0.786–0.951, *p* < 0.0001), 0.858 (95% CI 0.770–0.947, *p* < 0.0001), and 0.734 (95% CI 0.619–0.849, *p* = 0.001), respectively.

## Discussion

Ageing and longevity are one of the main concerns all over the world. In recent years, proteomics technology was widely used to identify age related plasma proteins, which can provide new clues about the mechanisms of aging process. Previous proteomic studies showed that most of age-relevant proteins were enriched in insulin-like growth factor (IGF) signaling, mitogen-activated protein kinases (MAPK), hypoxia-inducible factor 1 (HIF1), cytokine signaling, Forkhead Box O (FOXO) metabolic pathways, folate metabolism, advance glycation end products (AGE), and receptor AGE (RAGE) metabolic pathway [13]. Till now, few studies have focused on the relationship between blood glucose regulation and longevity. Regulation of blood glucose balance is part of the regulation of life activities. It is an important condition for maintaining homeostasis. When the body's blood glucose regulation is out of balance many diseases such as diabetes mellitus can be caused. According to the previous studies, we speculate that the longevity population may have some advantages in blood glucose regulation. In this study, we utilized TMT-based proteomics method to analyze the differences of plasma proteomics profiles between non-longevity area participants (with exceeding standard FUN level) and offsprings of longevous families (with normal FUN level). In total, we identified 155 DEPs (non-longevity area participants vs. offsprings of longevous families, 132 down-regulated and 23 up-regulated). According to bioinformatics analysis, several DEPs were enriched in glycolysis/gluconeogenesis, pyruvate metabolism, propanoate metabolism, fructose and mannose metabolism, pentose phosphate pathway, glucagon signaling pathway, PI3K-Akt signaling pathway, etc. (Additional file 8: Table S7). These are involved in processes of metabolism of carbohydrate and regulation of blood glucose concentration.

### Regulation of carbohydrate metabolism related proteins

Carbohydrate metabolism can be divided into catabolism and anabolism, mainly including aerobic oxidation, glycolysis, pentose phosphate pathway, gluconeogenesis andetc. It is well known that many enzymes play important roles in carbohydrate metabolism processes, such as aerobic oxidation enzymes in glycolysis and pentose phosphate pathway (e.g., triosephosphate isomerase (TPI), glyceraldehyde 3-phosphate dehydrogenase (GAPDH), enolase (ENO), phosphoglycerate kinase (PGK), pyruvate kinase (PK), fructose-bisphos-phate aldolase (ALDO), L-lactate dehydrogenase (LDH), malate dehydrogenase (MDH), glucose phosphate isomerase (GPI), transaldolase (TALDO), and enzymes in gluconeogenesis (e.g., MDH, PK, LDH) [14–16]. In the present study, both aerobic oxidation enzymes (ALDOA, TPI1, GAPDH, PGK1, ENO1, GPI and TALDO1) and gluconeogenesis-related enzymes (MDH1, PKM, LDHA, and LDHB) were up-regulated in the samples from Bama longevity hotspot. These results suggested that compared with the high FUN population in non-longevity areas, the offsprings of longevous families in Bama improved both the catabolism of carbohydrate (gluconeogenesis) and the anabolism of carbohydrate (aerobic oxidation, glycolysis, pentose phosphate pathways and etc.), thus promoting the metabolic process of glucose.

### Regulation of the production and utilization of blood glucose

The production and utilization of blood glucose are regulated by hormones such as insulin and glucagon. Insulin can suppress blood glucose levels by promoting the transformation of bloodstream glucose into glycogen, fat and other non-sugars, and inhibiting glucose production from the liver [17]. Glucagon is the counter-regulatory hormone to the hypoglycemic effects of insulin, and thus the increased plasma glucagon levels will result in increased hepatic glucose production by suppression of glycogenesis and glycolysis, and stimulating of glycogenolysis and gluconeogenesis [18].

The metabolic functions of insulin are mainly exerted by PI3K- Akt pathway in insulin cell signaling. The PI3K- Akt pathway mediates many of the metabolic actions of insulin via phosphorylation of key metabolic substrates such as glycogen synthase kinase-3 for glycogen synthesis [19–21]. Several researches have demonstrated that 14-3-3 isoforms (i.e., 14-3-3 Ɛ/YWHAE, 14-3-3 β/YWHAB, 14-3-3 γ/YWHAG, 14-3-3 η/YWHAH, 14-3-3 θ /YWHAQ, 14-3-3 ζ/YWHAZ, and 14-3-3 σ/SFN) interact with effectors (e.g., IRS-1, Raf-1, AS160/TBD1C4, and FOXO1) in the insulin signaling pathway and in glucose metabolism [20, 22–25]. Lim GE et al*.* found insulin sensitivity decreased in an YWHAZ gene knockout mice model [26, 27]. In present study, the result showed that the most conspicuously enriched protein domain was 14-3-3 domain, YWHAZ, YWHAB, YWHAG and YWHAE were higher in samples of offsprings from Bama longevity hotspot (with normal FUN level) than in non-longevity area participants (with exceeding standard FUN level), which indicated that compared with non-longevity area participants (with exceeding standard FUN level), insulin plays a stronger role in Bama participants (with normal blood glucose).

As a major Ca^2+^ binding protein in non-muscle cells, calmodulin (CaM) is activated by Ca^2+^ and then undergoes a conformational change which allowing it to activate numerous downstream targets [28]. In humans, CaM is encoded by three genes (CALM1, CALM2, and CALM3) [29]. Glucagon is secreted from pancreaticα-cells in response to low levels of blood glucose, and intracellular Ca^2+^ activity is required for glucagon secretion [30]. Many studies support the hypothesis that the glucagon receptor type 1 (GR1)/phospholipase C (PLC)/inositol-3, 4, 5-triphosphate (IP3)/Ca^2+^/CaM pathway is the predominant or exclusive signal for glucagon in vivo most of the time [31, 32]. Epstein et al*.* found glucagon was expressed increasingly in islet cells in a mouse model of islet β-cell CaM overexpression [33]. Similarly, in the present study, we found that CALM3 was up-regulated in the samples from Bama longevity hotspot (Fig. 5), which suggests the regulation of glucagon in Bama participants (with normal blood glucose) is stronger than that in non-longevity area participants (with exceeding standard FUN level).

### Regulation of inflammatory response related proteins

Type 2 diabetes was an inflammatory condition, which associated with increasing levels of acute phase inflammatory reactants in serum [34–38]. In our study, several inflammatory reactant proteins, e.g., C—reactive protein (CRP), serum amyloid A-2 protein (SAA2), complement factor H (CFH), scavenger receptor cysteine-rich type 1 protein M130 (CD163), and lipopolysaccharide-binding protein (LBP) were down-regulated in the samples from Bama longevity hotspot, while serum amyloid A-4 protein (SAA4) and plasma serine protease inhibitor (SERPINA5) were up-regulated. Among these proteins, CRP and serum amyloid A (SAA) are important representative acute phase inflammatory proteins. It is reported that CRP significantly increased in the presence of inflammation and the elevated CRP level was associated with insulin resistance and an increased risk of diabetes [39–41]. As another important acute inflammation protein, SAA helps to link the complex network of cells and proteins mediating inflammation. The SAA gene family contains four genes, namely SAA1, SAA2, SAA3 and SAA4. SAA1 and SAA2 are acute-phase proteins, while SAA3 is a non-translated pseudogene and SAA4 protein is not induced during the acute phase response of inflammation [42–44]. Our proteomic result revealed that CRP and SAA2 are higher in non-longevity areas participants (with exceeding standard FUN level) than in offsprings of longevous families (with normal FUN level), which is consistent with the aforementioned reports.

Besides the aforementioned proteins, some other proteins including SPARC, PARK 7, and IGFBP-1 were significantly down-regulated, whereas pantetheinase (VNN1) was significantly up-regulated in non-longevity area participants (with exceeding standard FUN level) (Figs. 5, 6, and Supporting Additional file 3: Table S2). Consistent with our result, it is reported that the SPARC levels were decreased in islets with diabetes and SPARC deficiency could lead to DM in SPARC null mice [45, 46]. On the contrary, Wu et al*.* reported that plasma SPARC levels were significantly increased in T2DM patients, and Xu et al*.* found that increased plasma SPARC levels were relevant to insulin resistance and dyslipidemia in gestational diabetes patients [47, 48]. Therefore, more studies are still needed for further validation of the mechanism of SPARC on glycemic control. Furthermore, DJ-1 gene (PARK7) was found to be down-regulated in pancreatic islets of patients with type 2 diabetes mellitus (T2DM) [49], and a low serum concentration of IGFBP-1 is associated with gestational diabetes mellitus (GDM), unfavorable metabolic profile, glucose intolerance and risk of diabetes mellitus [50, 51], which are similar to our results. In addition, consistent with our results, some studies reported blood levels of VNN1 were increased in diabetic patients, and VNN1 increased the expression of gluconeogenic genes and hepatic glucose output, which led to hyperglycemia in a diabetic mice model [52, 53]. Further research is needed to reveal the glycemic control mechanism of these DEPs.

## Conclusion

In summary, the present study investigated the global plasma proteomic changes of non-longevity area participants (with exceeding standard FUN level) and offspring of longevous families (with normal FUN level). The 155 identified DEPs were annotated in 33 GO functional groups, 18 COG categories, and 21 KEGG pathways. The DEPs related to metabolism of carbohydrate were mainly involved in glycolysis/gluconeogenesis, pyruvate metabolism and propanoate metabolism. Based on PPI network analysis, we found carbohydrate metabolism, PI3K-Akt signaling pathway, glucagon signaling pathway and inflammatory response were the crosstalk signaling clusters which contained most of the blood glucose regulation related DEPs. In addition to the common diabetes markers reported, we found that YWHAZ, YWHAB, YWHAG, YWHAE, CALM3, CRP, SAA2, PARK 7, IGFBP1 and VNN1 can be used as potential biomarkers for predicting abnormal blood glucose levels. Our research can give a better understanding on the potential mechanism of blood glucose regulation in longevity population and on the potential evaluation of candidate biomarkers or therapeutic targets of glycemic control.

## Supplementary Information

**Additional file 1: Figure S1.** Workflow chart of this research.

**Additional file 2: Table S1.** The detailed data of identified proteins.

**Additional file 3: Table S2.** Differentially expressed statistics of DEPs.

**Additional file 4: Table S3.** GO Terms Classify of DEPs.

**Additional file 5: Table S4.** Subcellular Classify of DEPs.

**Additional file 6: Table S5.** COG Classify of DEPs.

**Additional file 7: Table S6.** GO enrichment of DEPs.

**Additional file 8: Table S7.** Protein domain enrichment of DEPs.

**Additional file 9: Table S8.** KEGG_pathway enrichment of DEPs.

## Data Availability

The MS proteomics data have been deposited to Proteome X change with the dataset identifier PXD032932 (the reviewer account: reviewer_pxd032932@ebi.ac.uk; password: BovEry7u).

## Abbreviations

TG: Triglyceride
TC: Total cholesterol
HDLC: High density liptein cholesterol
LDLC: Low density liptein cholesterol
APOA1: Apolipoprotein A1
APOB: Apolipoprotein B
FUN: Fructosamine
DEPs: Differentially expressed proteins
DM: Diabetes mellitus
FPG: Fasting plasma glucose
2-hPG: The 2-h plasma glucose
OGTT: Oral glucose tolerance test
HbA1c: Glycated haemoglobin
1, 5-AG: 1, 5-Anhydroglucitol
IDF: International Diabetes Federation
TMT: Tandem mass tag
IBT: Institute of blood transfusion
FDR: False discovery rate
GO: Gene Ontology
COG: Clusters of Orthologous Groups
KEGG: Kyoto Encyclopedia of Genes and Genomes
PPI: Protein-protein interaction
SPARC: Secreted protein acidic and rich in cysteine
CD163: Scavenger receptor cysteine-rich type 1 protein M130
PARK 7: Protein/nucleic acid deglycase DJ-1
IGFBP-1: Insulin-like growth factor-binding protein 1
PI3K: Phosphatidylinositol 3-kinase
IGF: Insulin-like growth factor
MAPK: Mitogen-activated protein kinases
HIF1: Hypoxia-inducible factor 1
FOXO: Forkhead Box O
AGE: Advance glycation end products
RAGE: Receptor AGE
TPI: Triosephosphate isomerase
GAPDH: Glyceraldehyde 3-phosphate dehydrogenase
ENO: Enolase
PGK: Phosphoglycerate kinase
PK: Pyruvate kinase
ALDO: Fructose-bisphos-phate aldolase
LDH: L-lactate dehydrogenase
MDH: Malate dehydrogenase
GPI: Glucose phosphate isomerase
TALDO: Transaldolase
CaM: Calmodulin
GR1: Glucagon receptor type 1
PLC: Phospholipase C
IP3: Inositol-3, 4, 5-triphosphate
CRP: C-reactive protein
SAA2: Serum amyloid A-2 protein
CFH: Complement factor H
LBP: Lipopolysaccharide-binding protein
SAA4: Serum amyloid A-4 protein
SERPINA5: Plasma serine protease inhibitor
SAA: Serum amyloid A
VNN1: Pantetheinase
