# Supplementary figures and images for: Proteomics profiles of blood glucose-related proteins involved in a Chinese longevity cohort
Source: Clin Proteomics. 2022 Dec 3;19:45. doi: 10.1186/s12014-022-09382-w (PMC9719669; doi:10.1186/s12014-022-09382-w)

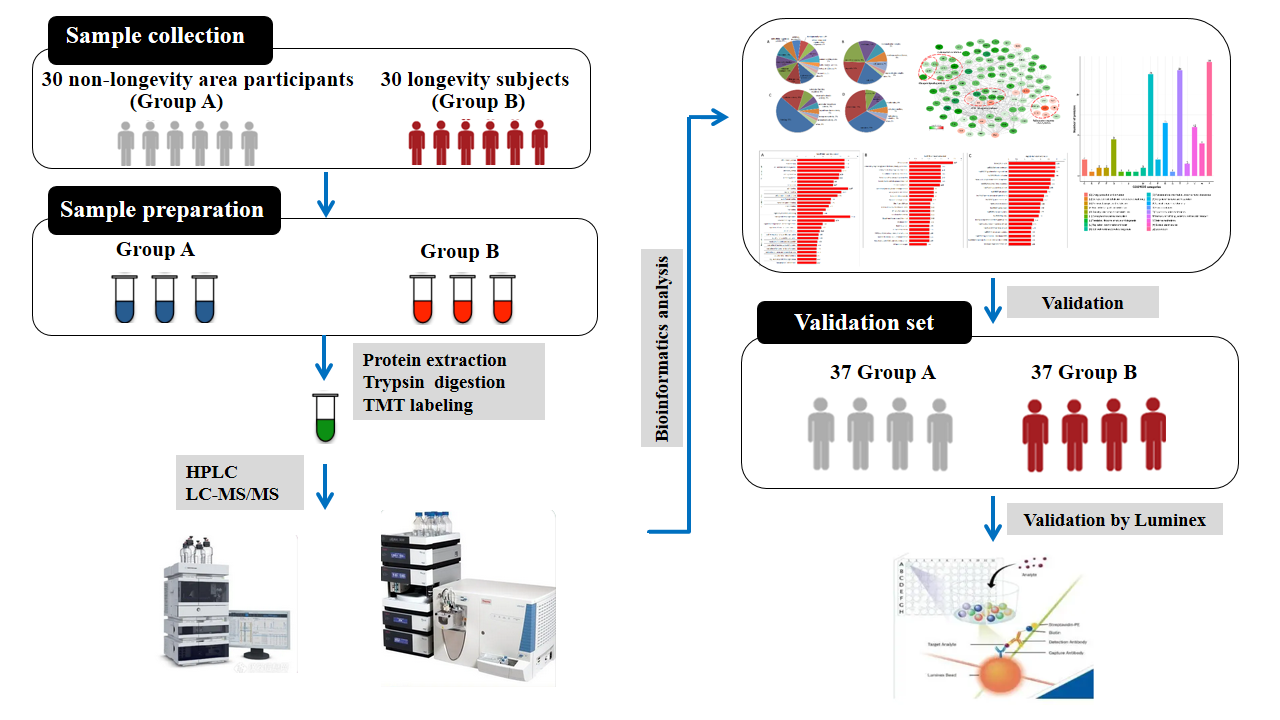

Supplement: Supplementary file 1 — Additional file 1: Figure S1. Workflow chart of this research. [file 12014_2022_9382_MOESM1_ESM.tif]
